# An Interpretable Method for Asphalt Pavement Skid Resistance Performance Evaluation Under Sand-Accumulated Conditions Based on Multi-Scale Fractals

**DOI:** 10.3390/s25102986

**Published:** 2025-05-09

**Authors:** Yuhan Weng, Zhaoyun Sun, Huiying Liu, Yingbin Gu

**Affiliations:** School of Information Engineering, Chang’an University, Xi’an 710064, China; wengyuhan@chd.edu.cn (Y.W.); 2023124008@chd.edu.cn (H.L.); 2022124139@chd.edu.cn (Y.G.)

**Keywords:** asphalt pavement, three-dimensional surface texture, skid resistance, wavelet transform, fractal, machine learning, interpretability

## Abstract

**Highlights:**

**What are the main findings?**

- The integration of a wavelet transform and fractal theory effectively extracts multi-scale fractal features of pavement texture, and the proposed model shows superior performance in skid resistance evaluation.
- An interpretable analysis reveals that the fractal dimension of texture in the 0.4~0.8 mm range most significantly affects model output.

**What are the implications of the main findings?**

- Multi-scale fractal texture features are effective indicators of skid resistance under sand-accumulated conditions, and the proposed model’s good performance in robustness experiments implies its potential for practical application in pavement engineering.
- This research innovatively applies an interpretable analysis method to the field of asphalt pavement skid resistance, providing inspiration for proposing more complex and accurate models.

**Abstract:**

The skid resistance of asphalt pavement is vital for traffic safety and reducing accidents. Existing research using only wavelet transforms or fractal theory to study the pavement surface texture-skid resistance relationship has limitations. This paper integrates a wavelet transform and fractal theory to extract the multi-scale fractal features of pavement texture. It proposes an interpretable machine learning model for skid resistance assessments of sand-accumulated pavements. The three-dimensional (3D) texture of asphalt pavements is decomposed at multiple scales, and fractal and multifractal features are extracted to build a dataset. The performance of mainstream machine learning models is compared, and the eXtreme Gradient Boosting (XGBoost) model is optimized using the Covariance Matrix Adaptation Evolution Strategy (CMA-ES) algorithm. The SHapley Additive exPlanations (SHAP) method is used to analyze the optimal model’s interpretability. The results show that asphalt concrete with a maximum nominal particle size of 13 mm (AC-13) has the most concentrated fractal dimension, followed by open-graded friction course with a maximum nominal particle size of 9.5 mm (OGFC-10), with stone matrix asphalt with a maximum nominal particle size of 16 mm (SMA-16) being the most dispersed. The singular intensity difference of the multifractal (Δ*α*) changes oppositely to the fractal dimension (*D*), and the fractal dimension difference of the multifractal (Δ*f*) decreases with the number of wavelet decomposition layers. The CMA-ES-XGBoost model improves R^2^ by 27.1%, 9%, and 3.4% over Linear Regression, Light Gradient Boosting Machine (LightGBM), and XGBoost, respectively. The 0.4–0.8 mm range fractal dimension most significantly impacts the model output, with complex interactions between features at different scales.

## 1. Introduction

In recent years, with the proposal of the Transportation Power policy, China has been vigorously promoting the coordinated development of transportation infrastructure, aiming to build a modern comprehensive transportation system that integrates safety, convenience, and intelligence. However, as road traffic continues to develop rapidly, ensuring road traffic safety has become an urgent problem in the transportation field. The skid resistance of the pavement is an important factor affecting road traffic safety and plays a crucial role in reducing vehicle accidents [1]. The skid resistance mainly depends on the friction generated between the tire and the pavement. Insufficient pavement skid resistance significantly increases the risk of traffic accidents.

The development of skid resistance between tires and road surfaces is a complex process influenced by multiple factors. Considering the entire contact process, three primary factors affect the skid resistance of pavements: the pavement itself, the tire, and the contact environment [2]. In practical environments, skid resistance performance is inevitably affected by external factors such as rain, snow, and ice, which change the friction characteristics between tires and pavement, thereby affecting skid resistance [3,4]. Especially in desert areas, sand accumulation poses a serious threat to the skid resistance of asphalt pavement by reducing the effective contact area and friction coefficient between tires and pavement [5].

The texture characteristics of the pavement surface are an important manifestation of the functional properties of the road surface and are also an important indicator affecting the skid resistance of the road [6,7]. In 1987, at the 17th World Road Congress organized by the World Road Association (PIARC) in Brussels, pavement texture was classified into four categories based on scale: micro-texture, which consists of microscopic convexities on the aggregate surface with a horizontal wavelength of less than 0.5 mm and a vertical amplitude of 1–50 μm, creating adhesion friction for vehicles; macro-texture, which refers to surface roughness with a horizontal wavelength of 0.5–50 mm and a vertical amplitude of 0.1–20 mm, generating hysteresis friction for vehicles; coarse texture; and unevenness [8]. Many scholars have conducted research on this topic. Kienle et al. [9] studied the effect of surface macro-texture on skid resistance under wet conditions and used a mathematical model to discuss the impact of these factors on skid resistance. Du et al. [10] used texture recognition and deep neural network algorithms to establish an association model between texture images and skid resistance. Xie et al. [11] studied the contributions of the macro-texture and micro-texture of asphalt pavement to skid resistance and used the Generalized Extreme Studentized Deviate (GESD) and the Neighboring-region Interpolation Algorithm (NRIA) to identify and replace outliers and suppress noise in texture data. Wang et al. [12] developed a wear-resistant ultra-thin wearing course and used the Tire–pavement Dynamic Friction Analyzer (TDFA) to conduct wear tests and analyze the relationship between the skid resistance and macro-texture of the wearing course. Dong et al. [13] studied the effect of the shape characteristics of coarse aggregates on the skid resistance of asphalt pavement and found that micro-texture has a greater impact on the skid resistance of actual asphalt pavement than the angularity of coarse aggregates. Yang et al. [14] introduced a new high-resolution 3D laser imaging technology for the non-contact continuous measurement of pavement performance, including its texture, friction, and hydroplaning speed. Li et al. [15], using 3D image technology, explored the texture indices applied to pavement wear analysis. Díaz-Torrealba et al. [16] further improved the post-construction roughness prediction model for asphalt overlay based on profile transformation. Chu et al. [17] proposed an improved 3D pavement texture reconstruction method based on interference fringes.

The relationship between multi-scale texture features and skid resistance has been a key research focus. The common practice is to classify pavement texture into macro- and micro-textures following PIARC’s definition. However, some researchers employ signal decomposition techniques for a more refined classification of pavement texture. They aim to investigate the relationship between pavement texture and skid resistance at a more detailed scale. Yang et al. [18] used wavelet transform to map macro-texture depth features to different wavelength regions and found that the texture with a maximum wavelength of 3.2 mm and located in the top 2.5 mm is the key contact area for pavement–tire interactions. Yu et al. [19] studied the effect of Continuous Friction Measurement Equipment (CFME) on test sites with different preventive maintenance treatments and developed a statistical friction model using texture parameters extracted by Hilbert–Huang Transformation (HHT). Li et al. [20] proposed a method based on 2D wavelet transform to characterize the micro-texture and macro-texture of asphalt pavement.

Currently, many scholars are studying the relationship between the fractal characterization of pavement texture and skid resistance. Liu et al. [21] hierarchically deconstructed pavement texture and explored the fractal characteristics of each layer to determine the texture layer with the greatest impact on skid resistance. Miao et al. [22] studied the fractal and multifractal properties of the macro-texture on asphalt pavement and found strong correlations between the fractal dimension (*D*), horizontal multifractal spectrum difference (Δ*α*), vertical multifractal spectrum difference (Δf(*α*)), and mean texture depth (MTD) and the test value of a Dynamic Friction Tester at 60 km/h (DFT60). Ran et al. [23] proposed a new method based on digital image processing and morphology to evaluate asphalt pavement segregation by analyzing the multifractal properties of pits in binary images.

With the advent of the big data era, machine learning has been widely applied in various fields such as medical diagnosis, financial risk assessment, and autonomous driving. The interpretability of machine learning models has become a key concern. However, complex models like deep learning are often seen as “black boxes”, leading to issues such as uncertainty in medical diagnoses and difficulty in explaining high-risk financial decisions. Similarly, in pavement engineering, the “black box” nature of machine learning models causes problems like uncertain maintenance decisions, hindered model optimization, reduced user trust, and increased safety risks. These issues restrict the effective application of machine learning in pavement engineering, making the improvement of model interpretability crucial for the field’s development. In recent years, interpretability research has gained attention, with methods like feature importance assessment, Local Interpretable Model-agnostic Explanations (LIME) [24], and SHapley Additive exPlanations (SHAP) [25] emerging.

The current research shows that 3D morphology-based fractal characterization is rapidly developing, and various simple yet powerful fractal algorithms are being used to process pavement texture information. The fractal dimension has good capabilities in texture characterization and skid resistance evaluation. However, assessing the correlation between the fractal dimension and skid resistance often relies on statistical indicators like the mean profile depth (MPD) and MTD. Traditional skid resistance testing equipment has unstable results, and the MPD is insufficient to characterize skid resistance [26].

To address these issues, this paper combines a wavelet transform and fractal theory to extract fractal features representing the self-similarity of the pavement texture at multiple scales and proposes an interpretable machine learning model based on multi-scale fractal features for the intelligent assessment of skid resistance on sand-accumulated pavements. First, the 3D texture of the asphalt pavement is decomposed at multiple scales, and fractal and multifractal dimension features are extracted at each scale to build a multi-scale fractal feature dataset. Then, the predictive performance of mainstream machine learning models is compared, and the CMA-ES algorithm is used to optimize the best-performing XGBoost model. Finally, the SHAP method is employed for an interpretability analysis of the optimal model. This study provides new insights into understanding how pavement texture affects skid resistance and how multi-scale fractal features influence model decisions.

## 2. Materials and Methods

**Figure 1:**
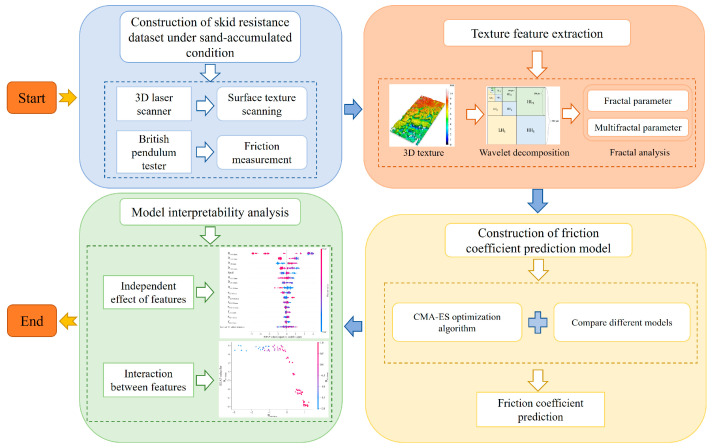
Flowchart of this study.

As shown in Figure 1, the flowchart of this interpretable skid resistance evaluation study is divided into four main parts, each with specific tasks and objectives. The first step is to construct a skid resistance dataset under sand-accumulated conditions. This involves using a 3D laser scanner to capture surface texture and a pendulum friction meter to measure friction coefficients. Next, texture features are extracted through a wavelet transform and fractal analysis to obtain detailed features like fractal and multifractal parameters. Then, a friction coefficient prediction model is developed by comparing and selecting the best evaluation model, which is then fine-tuned using the CMA-ES optimization algorithm. Finally, the model interpretability is analyzed. This includes assessing the individual impact of each feature on the model output and how features interact to influence predictions. By analyzing the model’s interpretability, we can understand its decision-making process and identify the most influential features.

### 2.1. Materials and Data Collection

To explore the multi-scale fractal properties of different asphalt mixtures, this study prepared specimens of different standard gradation types, such as dense-graded asphalt concrete (AC), stone matrix asphalt (SMA), and open-graded friction course (OGFC). As per China’s current “Highway Asphalt Pavement Design Specifications” (JTG D50-2017) [27], three test specimens sized 300 × 300 × 50 mm were designed and produced, as detailed in Table 1. Limestone served as the aggregate, and SBS-modified asphalt was utilized. The basic indices of the asphalt binder and aggregates were tested according to the “Standard Test Methods of Bitumen and Bituminous Mixture for Highway Engineering” (JTG E20-2011) [28], and the results are shown in Table 2 and Table 3. The optimal bitumen contents were 4.8%, 4.9%, and 5.4%, respectively, for AC-13, OGFC-10, and SMA-16 using the Marshall design method. The distribution of sand particles is shown in Table 4.

#### 2.1.1. Friction Coefficient Test

**Figure 2:**
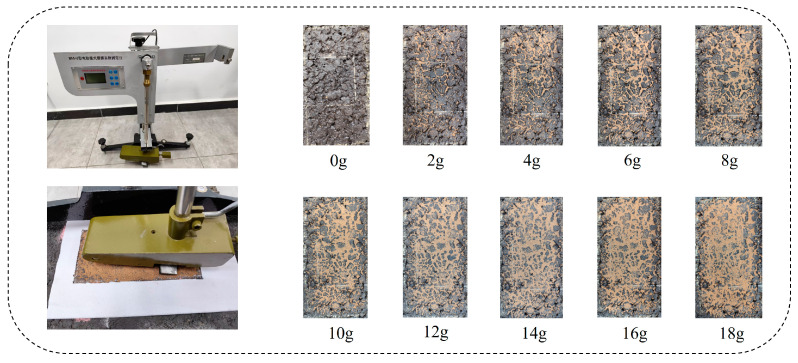
Friction coefficient test on sand-accumulated specimens.

The British Pendulum Tester (BPT) is used to evaluate the skid resistance of road surfaces by simulating the interaction between tires and pavement. In this study, a digital BPT with an integrated digital measurement system, featuring precise angle sensors and automated control programs, was employed as shown in Figure 2. The British Pendulum Number (BPN), measured when the BPT’s pendulum reaches its highest point, reflects the skid resistance of the pavement.

To measure skid resistance under sand-accumulated conditions, sand of different volumes was evenly spread in the test area to simulate such conditions. The sand content per unit area was calculated using Equation (1):

SandCon = Mess/Area,(1)

where SandCon (g/cm^2^) is the sand content per unit area, Mess (g) is the mass of sand (increasing by 2 g each time), and Area (cm^2^) is the size of the test area (20 cm × 10 cm). In the test area of the specimen, following the current Chinese standard “Field Test Methods of Highway Subgrade and Pavement” (JTG 3450-2019) [29], with an air temperature of 25 °C and humidity of 50%, we used the BPT for continuous measurement, recorded the BPN five consecutive times, and calculated the mean value ${BPN}_{mean}$. For each specimen, several sets of friction data were collected under clean and different sand-accumulated conditions. The experiment stopped when the ${BPN}_{mean}$ stabilized within ±2 after the addition of more sand. In total, 138 sets of friction data were collected for all gradation types.

#### 2.1.2. Three-Dimensional Texture Data Acquisition and Processing

**Figure 3:**
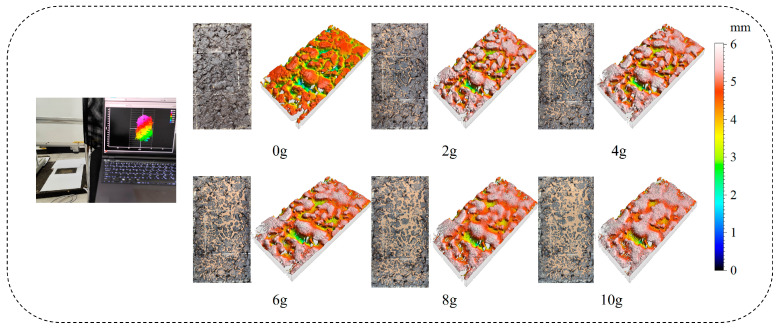
Three-dimensional texture acquisition.

This study employed the Gocator 3110 3D intelligent sensor by LMI, Mississauga ON, Canada, to precisely measure the surface texture of specimens. As shown in Figure 3, the Gocator 3110, featuring binocular stereo vision, was mounted on a horizontal metal bracket. Its X/Y axis scanning interval was set at 0.1 mm, with a vertical resolution of 0.006 mm. After a specimen was placed within the scanning range, the high-precision measurement captured detailed texture information.

**Figure 4:**
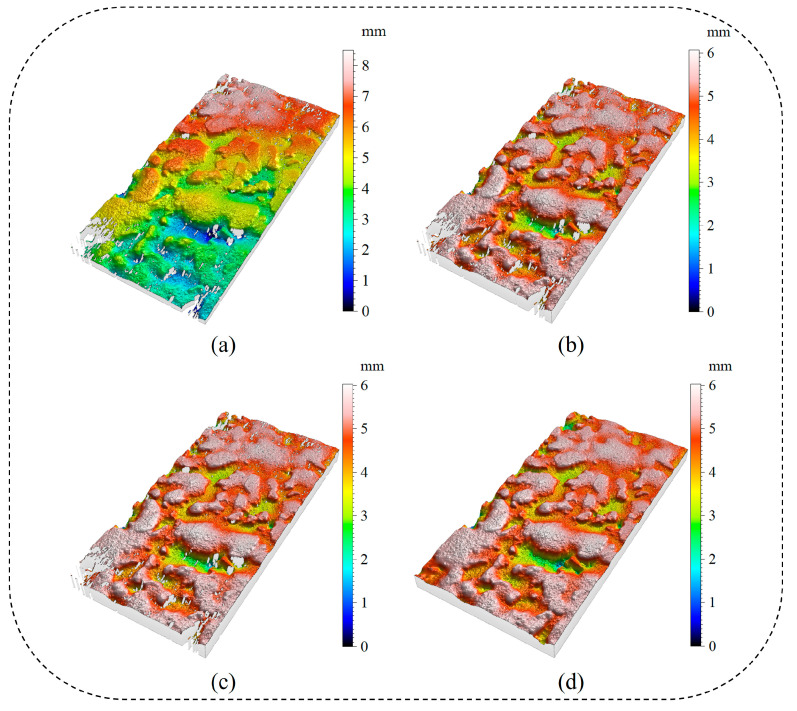
Preprocessing of point cloud data. (**a**) raw point cloud; (**b**) point cloud calibration; (**c**) point cloud denoising; and (**d**) point cloud completion.

To enhance the accuracy of the raw point cloud data (Figure 4a), three preprocessing steps were implemented: calibration (Figure 4b), denoising (Figure 4c), and completion (Figure 4d), yielding higher-quality data for subsequent feature extraction.

### 2.2. Methods

#### 2.2.1. Two-Dimensional Wavelet Transform

In this study, two-dimensional wavelet transform technology [30] was used to analyze the pavement texture. It decomposes the pavement texture into sub-textures at different scales. This method allows each sub-texture to contain texture features of specific wavelengths, providing a powerful tool for understanding the relationship between road friction properties and textures at specific scales.

**Figure 5:**
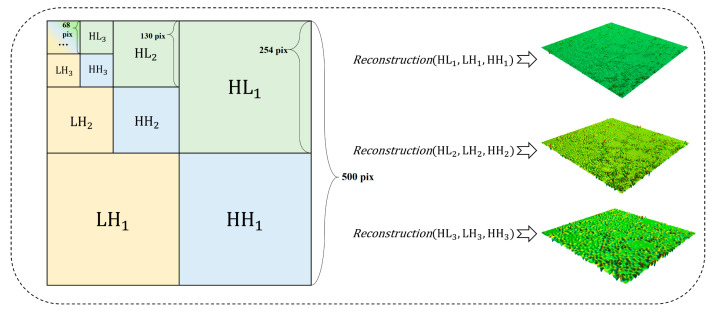
Decomposition and reconstruction of pavement texture.

A wavelet transform enables the analysis of textures with varying degrees of fineness at different levels. As shown in Equation (2), the sum of the high-frequency coefficients in different directions gives ${Level}_{i}$, the high-frequency wavelet coefficient for each level (the low-frequency coefficients of each level are further decomposed):

(2) $${\mathrm{Level}}_{i}={\mathrm{HL}}_{i}+{\mathrm{LH}}_{i}+{\mathrm{HH}}_{i},$$

where i denotes the level. As shown in Figure 5, each decomposition yields a set of low-frequency coefficients and three sets of high-frequency coefficients, representing low-frequency (LL), horizontal high-frequency (HL), vertical high-frequency (LH), and diagonal high-frequency (HH) texture features. The high-frequency information is used to reconstruct sub-textures at different scales. For example, the Level 1 sub-texture is reconstructed using the high-frequency information ${\mathrm{HL}}_{1},\;{\mathrm{LH}}_{1},\;\mathrm{and}\;{\mathrm{HH}}_{1}$ (denoted by $L_{1}$). As shown in Figure 5, with an increase in the decomposition level, the reconstruction results generally evolve from describing texture details to outlining texture contours.

#### 2.2.2. Fractal Theory

The fractal dimension, a key index for fractal patterns, quantifies complexity as the ratio of detail change to scale change. It is mainly divided into fractals and multifractals [22]. Fractals describe the overall and average characteristics of an object. To study them, the fractal dimension *D* is introduced as a quantification index. The most common method for calculating a fractal is the box-counting method, defined as Equation (3):

(3) $$D=\underset{\epsilon \to 0}{\lim}\frac{\log\mathrm{Num}(\epsilon )}{\log(1/\epsilon )},$$

Here, Num(ϵ) is the number of boxes, and ϵ is the box size.

Multifractals, on the other hand, depict the local singularity and inhomogeneity of an object’s surface. This theory offers richer details than fractals. The calculations for the multifractal spectrum are shown in Equation (4):

(4) $$\left\{\begin{array}{c} \alpha (q)=d\tau (q)/d(q)\\ f(q)=q\alpha (q)-\tau (q) \end{array}\right.,$$

In these formulas, α(q) is the singularity intensity with *q* as the independent variable; f(q) is the fractal dimension with *q* as the independent variable; and τ(q) is the mass exponent of the q-order moment. The functional relationship between f(α) and α is recognized as the multifractal spectrum. Commonly used indicator parameters for the multifractal spectrum are defined in Equations (5) and (6):

(5) $$∆\alpha =\alpha_{\max}-\alpha_{\min},$$

(6) $$∆f(\alpha )=f\left(\alpha_{\min}\right)-f(\alpha_{\max}),$$

#### 2.2.3. Multi-Scale Fractal Feature Extraction

Using wavelet transforms or fractal theory alone to analyze the relationship between pavement texture and skid resistance has limitations. (1) A wavelet transform achieves multi-scale analysis but cannot quantify finer texture features. (2) Fractal theory considers the randomness and self-similarity of the pavement texture, but using only the fractal dimension and multifractal spectrum parameters to characterize macro- and micro-textures is one-sided without decomposing the original data.

In this study, 2D wavelet transform was used to analyze the texture of point cloud data. The “sym4” wavelet, with four vanishing moments and good smoothness, was chosen for the multi-scale decomposition. This helps in capturing local singularities and reducing edge-related errors.

The 2D wavelet transform decomposed the original data into nine levels, each representing different spatial resolutions. Given the 0.1 mm X/Y resolution of the 3D point cloud data, each wavelet transform doubled the input resolution. After the wavelet transforms, texture data from 0.1 mm to over 25.6 mm were obtained. These levels (0.1~0.2 mm, 0.2~0.4 mm, 0.4~0.8 mm, 0.8~1.6 mm, 1.6~3.2 mm, 3.2~6.4 mm, 6.4~12.8 mm, 12.8~25.6 mm, and >25.6 mm) covered microscopic to macroscopic textures, offering a comprehensive analysis framework.

At each of the nine scales, fractal and multifractal dimensions were extracted to describe the texture complexity, yielding 27 features in total, as shown in Table 5.

#### 2.2.4. Interpretability for Machine Learning Models

SHAP, based on the Shapley value theory from cooperative game theory, fairly allocates the total payout among participants in machine learning model interpretation [23]. Here, each feature is a “participant”, and the model’s prediction is the “total payout”.

For a sample X with M features, the SHAP value measures each feature’s contribution to the model’s prediction. For machine learning models, especially ensemble ones, tree-based SHAP value calculation is commonly used. If the model’s prediction function is func(X), where $X=(x_{1},x_{2},...,x_{M})$, then the SHAP value $\phi_{i}$ for the i-th feature can be computed using Equation (7):

(7) $$\phi_{i}=\sum_{S\subseteq M_{i}}\frac{|S|!(M-|S|-1)!}{M!}[\mathrm{func}(X_{S}\cup \{X_{i}\})-\mathrm{func}(X_{S})],$$

where S is a subset of features excluding the i-th one, $X_{S}$ is the sample with only the features in S, and ∣S∣ is the size of S. This formula measures the average marginal contribution of feature $x_{i}$ across all feature combinations.

In tree-based SHAP calculations, feature interactions are also considered. The interaction between features i and j is defined as Equation (8):

(8) $$\Phi_{i,j}(\mathrm{func},X)=\sum_{S\subseteq M_{i,j}}\frac{|S|!(M-|S|-2)!}{2(M-1)!}\nabla_{i,j}(\mathrm{func},X,S),$$

where $\nabla_{i,j}(\mathrm{func},X,S)={\mathrm{func}}_{X}(S\cup \{i,j\})-{\mathrm{func}}_{X}(S\cup \{j\})-[{\mathrm{func}}_{X}(S\cup \{i\}-{\mathrm{func}}_{X}(S))]$, and $\Phi_{i,j}(\mathrm{func},X)$ represents the combined impact of features i and j on the prediction.

The total SHAP value for a feature encompasses both its individual effect and interactions with other features, as defined in Equation (9):

(9) $$\Phi_{i}(\mathrm{func},X)={\;\phi }_{i}+\sum_{j\neq i}\Phi_{i,j}(\mathrm{func},X),$$

Here, $\phi_{i}$ is the individual SHAP value of feature i, and $\Phi_{i,j}(\mathrm{func},X)$ is the interaction SHAP value between features i and j.

#### 2.2.5. Mainstream Machine Learning Models

XGBoost (eXtreme Gradient Boosting) [31] is a gradient-boosting-based machine learning algorithm that enhances prediction accuracy by integrating multiple decision trees. It iteratively constructs new trees to fit the residuals of previous models, incrementally boosting model performance.

The objective function is defined in Equation (10):

(10) $$\mathrm{Obj}=\underset{\theta }{\min}\sum_{i=1}^{n}L(y_{i},{\hat{y}}_{i})+\sum_{k=1}^{K}\Omega (f_{k}),$$

where $L(y_{i},{\hat{y}}_{i})$ represents the loss function, quantifying the discrepancy between predicted and actual values. $\Omega (f_{k})$ is a regularization term that controls model complexity and mitigates overfitting. *K* denotes the number of decision trees.

LightGBM (Light Gradient Boosting Machine) [32], developed by Microsoft, is a gradient-boosting decision tree algorithm that refines XGBoost’s tree-building process. It employs a histogram-based method to rapidly identify optimal segmentation points, reducing computational and memory demands. Its objective function mirrors XGBoost’s, optimizing the model by minimizing the loss function and regularization term.

Both XGBoost and LightGBM excel in handling large-scale datasets and are versatile in solving classification and regression problems.

## 3. Results

### 3.1. BPN Measurement Results

**Figure 6:**
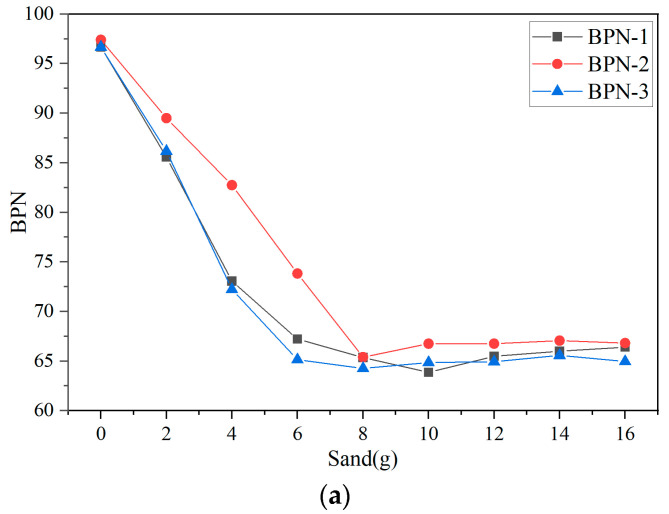

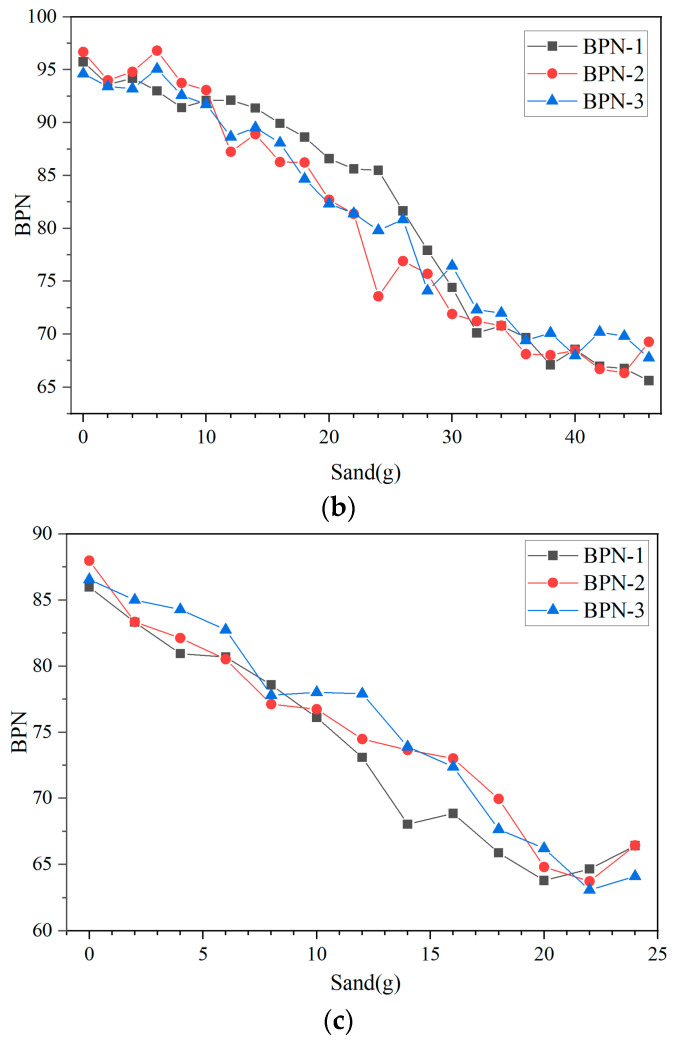
Measured BPN under sand-accumulated conditions, (**a**) AC-13; (**b**) OGFC-10, and (**c**) SMA-16.

As shown in Figure 6a–c, for AC-13, OGFC-10, and SMA-16, the BPN tended to stabilize once the sand accumulation in the test area reached 8 g (0.4 kg/m^2^), 38 g (1.9 kg/m^2^), and 22 g (1.1 kg/m^2^), respectively. From the data in Figure 6, it can be seen that when the surface began to accumulate sand, the measured BPN on AC-13 dropped sharply. In contrast, for OGFC-10 and SMA-16, the BPN exhibited an initial slow decline, followed by a rapid decrease, and finally stabilized.

### 3.2. Dataset Comparison

To compare the evaluation of skid resistance performance between the traditional texture features and the multi-scale fractal features proposed in this paper, 26 traditional texture features were extracted from the macro- and micro-textures to construct a dataset, as shown in Table 6. The XGBoost model was trained, and each dataset’s testing performance is shown in Table 7.

### 3.3. Model Comparison

The dataset was split into training and test sets in a 3:1 ratio to ensure independent model training and evaluation. Model performance was assessed on the test set using generalization errors: the root-mean-square error (RMSE) and mean absolute error (MAE) and the R^2^ score (coefficient of determination).

Optuna’s [33] CMA-ES hyperparameter optimization method was used to tune the XGBoost model on our custom dataset, with the final hyperparameters shown in Table 8.

Our proposed CMA-ES-XGBoost model outperformed traditional models like Linear Regression, LightGBM, and XGBoost, as shown in Table 9. Specifically, we observed the following:

- The RMSE was reduced by 36.5% compared to Linear Regression, 20.3% compared to LightGBM, and 9.8% compared to XGBoost;
- The MAE was lowered by 40.1% relative to Linear Regression, 16.6% relative to LightGBM, and 10.2% relative to XGBoost;
- The R^2^ value improved by 27.1% over Linear Regression, 9% over LightGBM, and 3.4% over XGBoost.

These results demonstrate that CMA-ES-XGBoost, with its optimized hyperparameters, has enhanced prediction accuracy and generalization ability.

**Figure 7:**
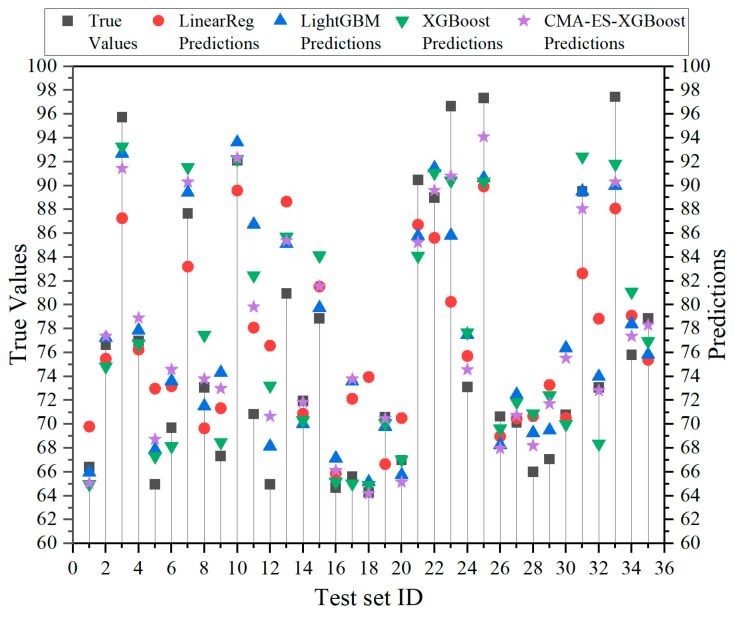
Comparison of test set results.

Figure 7 presents a scatter plot comparing predicted vs. actual values on the test set for different models. The x-axis shows test sample indices, and the y-axis shows predicted and actual values. While all models’ predictions clustered around the true values, CMA-ES-XGBoost showed the closest alignment in many cases, indicating superior prediction performance. The highest R^2^ showed that the predicted value of CMA-ES-XGBoost on the test set was generally nearest to the actual value.

## 4. Discussion

### 4.1. Analysis of Multi-Scale Fractal Features

**Figure 8:**
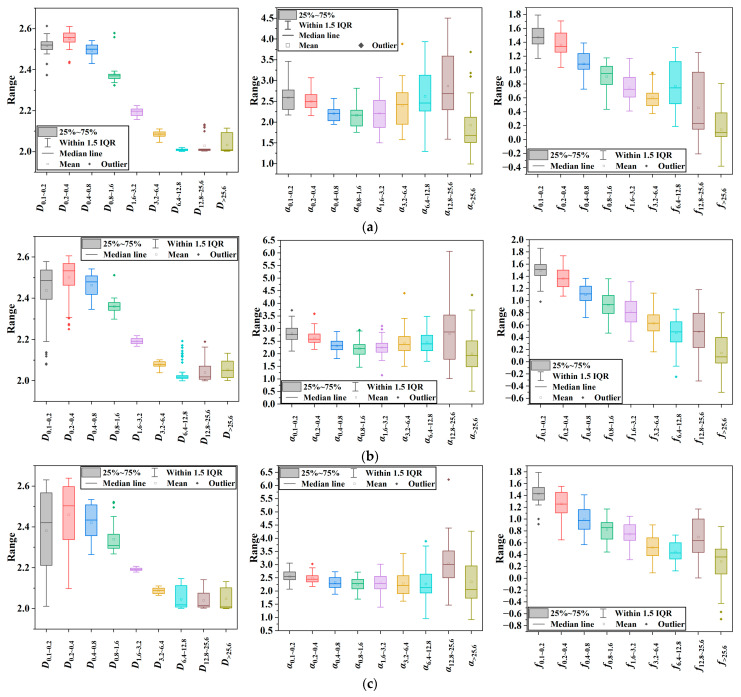
Variations in fractal and multifractal features at different scales. (**a**) AC-13; (**b**) OGFC-10; and (**c**) SMA-16.

As shown in Figure 8, the box plots of the fractal dimension *D* for different gradation types reveal that AC-13 has the most concentrated texture fractal dimensions, followed by OGFC-10, while SMA-16 has the most dispersed. This is because AC-13, a dense-graded asphalt mixture, has less variation in its surface texture at different decomposition levels, leading to a concentrated fractal dimension. In contrast, SMA-16, with its interrupted gradation, has more significant changes in its surface texture. The mean value of *D* first rises and then falls towards 2 with increasing decomposition level, indicating that larger-scale features are captured at lower levels and smaller-scale features at higher levels. Over-decomposition at higher levels can cause the fractal dimension to drop near 2, signaling a more uniform surface.

Observing the variation patterns of the multifractal dimensions ∆α and ∆f for different gradation types, it is evident that ∆α changes inversely to the fractal dimension *D*. Specifically, AC-13 has the highest box plot, OGFC-10 is in between, and SMA-16 has the lowest. For ∆f, its value generally decreases with increasing decomposition level across all three gradations. This indicates that as decomposition progresses, the structural characteristics of the asphalt mixture become more clearly distributed across scales, leading to a decrease in ∆f and reflecting increased homogeneity of the mixture’s structure across scales.

### 4.2. Interpretability Analysis and Discussion

**Figure 9:**
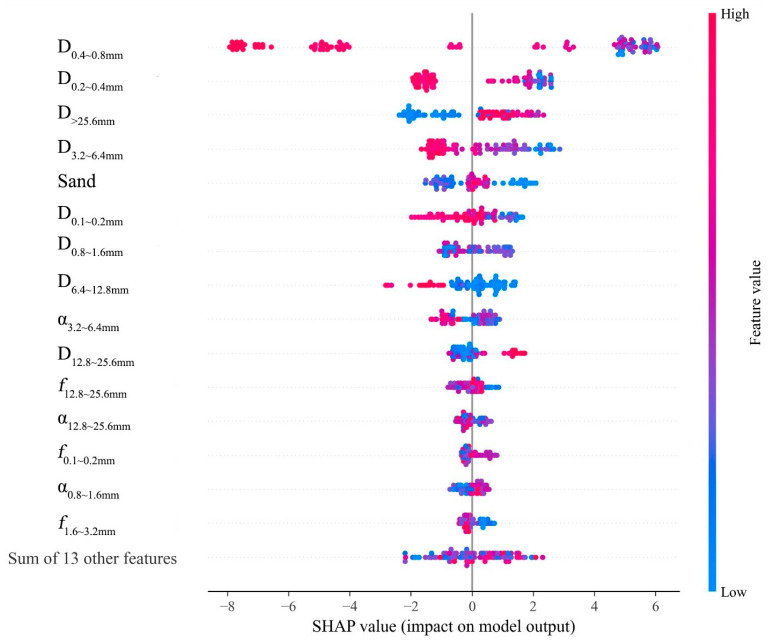
The impact of input features on the BPN.

Figure 9 is a scatter plot of SHAP values, visually presenting each feature’s impact on the model output (BPN). Each point represents a sample’s SHAP value, with the color intensity indicating the feature magnitude—red for high values and blue for low. The x-axis shows SHAP values, where positive values increase the prediction and negative ones decrease it. The y-axis lists the features involved in prediction, including $D_{0.4\sim 0.8\mathrm{mm}}$, $D_{0.2\sim 0.4\mathrm{mm}}$, $D_{>25.6\mathrm{mm}}$, $D_{3.2\sim 6.4\mathrm{mm}}$, Sand, and the “Sum of 13 other features”. The latter aggregates the SHAP values of less influential features.

In Figure 9, the position of each point reflects a sample’s SHAP value for a specific feature. For instance, low $D_{>25.6\mathrm{mm}}$ values (blue) are mostly on the left, decreasing the prediction. Conversely, if a feature’s high values (red) are on the right and its low values are on the left, the prediction increases with the feature value. High $D_{0.2\sim 0.4\mathrm{mm}}$ values are mostly on the left, reducing the prediction. $D_{3.2\sim 6.4\mathrm{mm}}$ and Sand have SHAP values spread across the axis, indicating complex effects on predictions.

By examining SHAP value distributions, key features for model predictions are identified. $D_{0.4\sim 0.8\mathrm{mm}}$ has the widest SHAP value range, stretching from left to right, showing the most significant impact on model output. This wide distribution means that $D_{0.4\sim 0.8\mathrm{mm}}$ greatly affects predictions, either increasing or decreasing them.

Furthermore, features like $\alpha_{12.8\sim 25.6\mathrm{mm}}$, $f_{0.1\sim 0.2\mathrm{mm}}$, $\alpha_{0.8\sim 1.6\mathrm{mm}}$, and $f_{1.6\sim 3.2\mathrm{mm}}$ have concentrated SHAP values, indicating consistent impacts across samples. In contrast, features with dispersed SHAP values, such as $D_{0.2\sim 0.4\mathrm{mm}}$, $D_{>25.6\mathrm{mm}}$, $D_{3.2\sim 6.4\mathrm{mm}}$, Sand, and $D_{0.1\sim 0.2\mathrm{mm}}$, show varying effects due to complex relationships or interactions with other variables.

**Figure 10:**
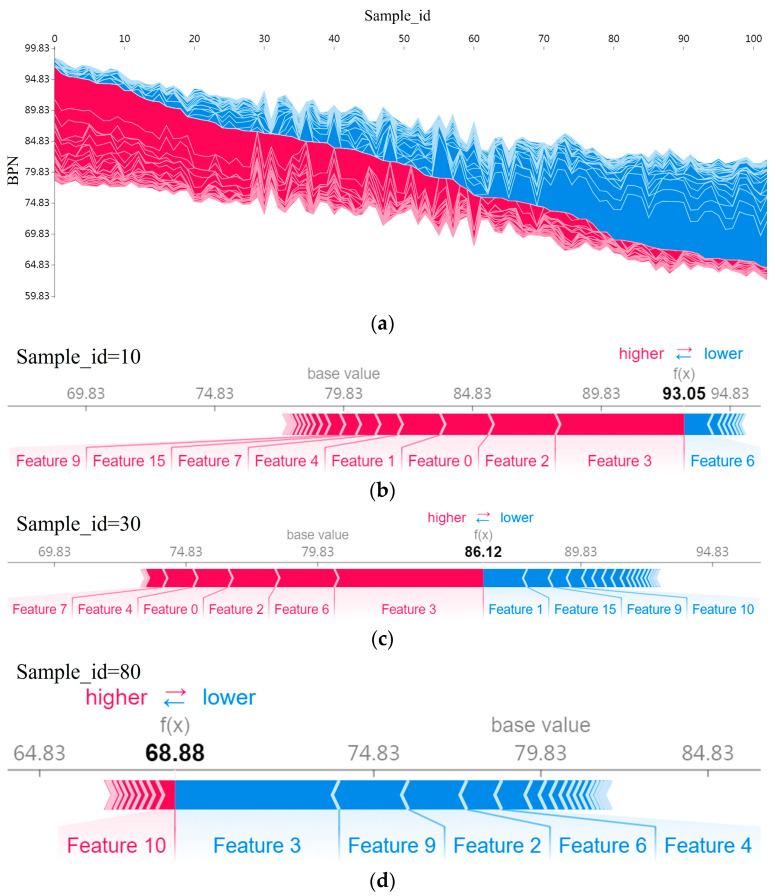
Force plot. (**a**) Force plot for all train set samples(red for positive contribution and blue for negative contribution); (**b**) detailed force plot example for sample with high BPN value; (**c**) detailed force plot example for sample with middle BPN value; and (**d**) detailed force plot example for sample with low BPN value.

Figure 10a is a force plot illustrating feature importance in predicting the BPN. The x-axis shows sample index IDs, sorted by target value from left to right. The y-axis indicates the target value (BPN). Colors represent feature contributions—red for positive and blue for negative, with the intensity reflecting the contribution magnitude.

From Figure 10, it is evident that different features dominate in different BPN ranges. Features contributing to higher predictions are more impactful when the BPN is large (Figure 10b). As the BPN decreases (Figure 10c,d), positive contributors diminish while negative ones increase, shown by the color shift from red to blue.

Notably, “Feature 2” and “Feature 3” ($D_{0.2\sim 0.4\mathrm{mm}}$ and $D_{0.4\sim 0.8\mathrm{mm}}$) frequently contribute, indicating their critical roles in predictions. Other features like “Feature 0”, “Feature 4”, and “Feature 6” (Sand, $D_{0.8\sim 1.6\mathrm{mm}}$, and $D_{3.2\sim 6.4\mathrm{mm}}$) also show varying contributions across BPN ranges.

**Figure 11:**
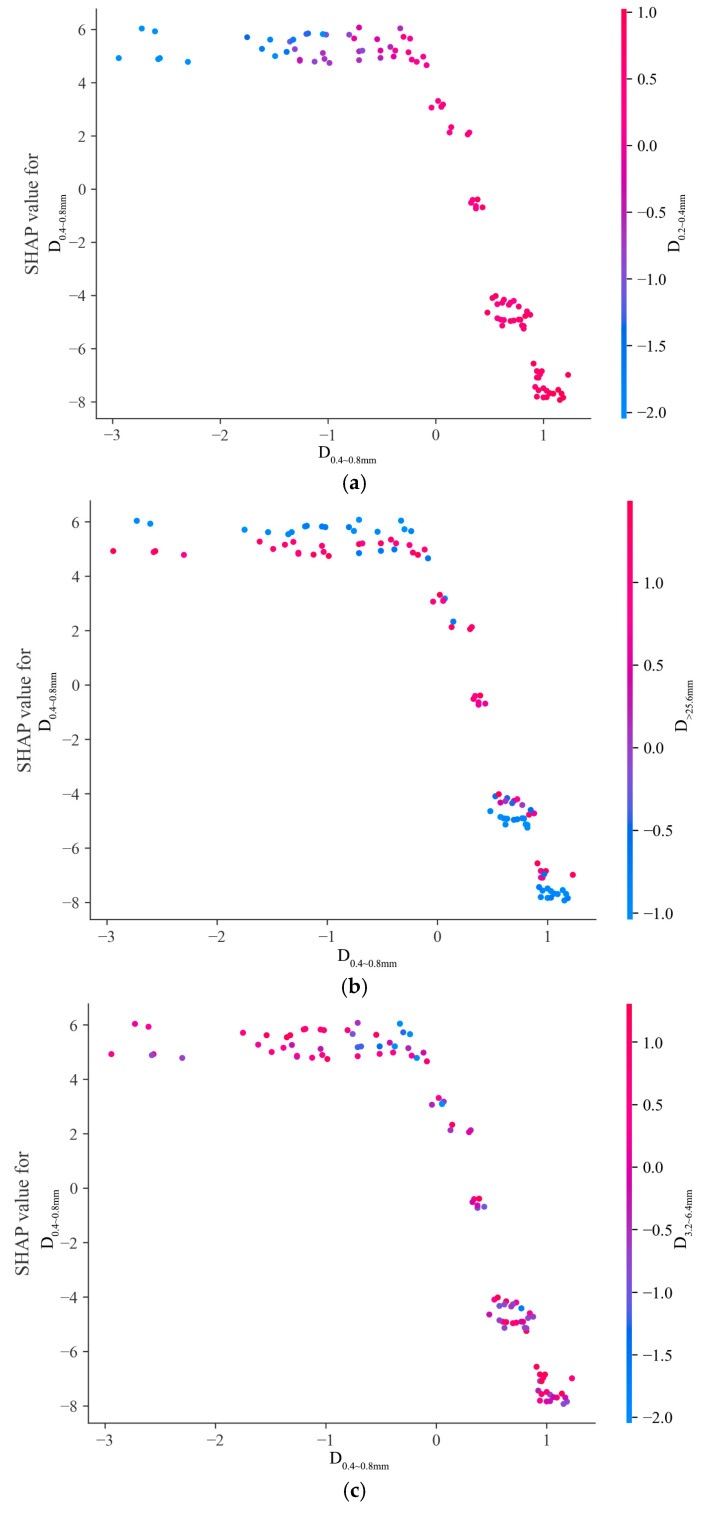

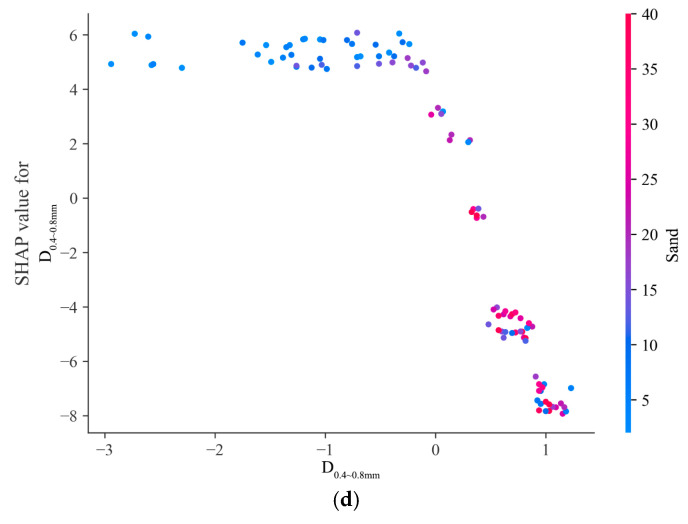
Feature interaction plots. (**a**) interactions between $D_{0.4\sim 0.8\mathrm{mm}}$ and $D_{0.2\sim 0.4\mathrm{mm}}$; (**b**) interactions between $D_{0.4\sim 0.8\mathrm{mm}}$ and $D_{>25.6\mathrm{mm}}$; (**c**) interactions between $D_{0.4\sim 0.8\mathrm{mm}}$ and $D_{3.2\sim 6.4\mathrm{mm}}$; and (**d**) interactions between $D_{0.4\sim 0.8\mathrm{mm}}$ and Sand.

Since $D_{0.4\sim 0.8\mathrm{mm}}$ significantly impacts the model output, its interaction with other features is analyzed in Figure 11, which shows how $D_{0.4\sim 0.8\mathrm{mm}}$ and four other key features ($D_{0.2\sim 0.4\mathrm{mm}}$, $D_{>25.6\mathrm{mm}}$, $D_{3.2\sim 6.4\mathrm{mm}}$, and Sand) jointly affect BPN predictions. Each subplot’s x-axis represents $D_{0.4\sim 0.8\mathrm{mm}}$ values, the left y-axis shows its SHAP values, and color indicates another feature’s value.

In Figure 11a, when $D_{0.2\sim 0.4\mathrm{mm}}$ is high, $D_{0.4\sim 0.8\mathrm{mm}}$ SHAP values tend to be negative, reducing predictions. When $D_{0.2\sim 0.4\mathrm{mm}}$ is low, $D_{0.4\sim 0.8\mathrm{mm}}$ SHAP values are positive, enhancing predictions. This reveals an interaction between $D_{0.4\sim 0.8\mathrm{mm}}$ and $D_{0.2\sim 0.4\mathrm{mm}}$ affecting model output.

Figure 11b,c depict the interactions of $D_{0.4\sim 0.8\mathrm{mm}}$ with $D_{>25.6\mathrm{mm}}$ and $D_{3.2\sim 6.4\mathrm{mm}}$, respectively. Changes in $D_{>25.6\mathrm{mm}}$ and $D_{3.2\sim 6.4\mathrm{mm}}$ values alter $D_{0.4\sim 0.8\mathrm{mm}}$ SHAP values, indicating complex impacts. Different value combinations change the direction and strength of $D_{0.4\sim 0.8\mathrm{mm}}$’s impact, showing that the model considers these feature interactions when predicting the BPN.

Figure 11d illustrates the interaction between $D_{0.4\sim 0.8\mathrm{mm}}$ and Sand. High Sand values correlate with negative $D_{0.4\sim 0.8\mathrm{mm}}$ SHAP values, indicating a negative impact. Low Sand values result in a broader but mostly positive SHAP value distribution for $D_{0.4\sim 0.8\mathrm{mm}}$, highlighting its stronger positive influence when Sand is low.

## 5. Conclusions

This paper combined wavelet transforms and fractal theory to extract the multi-scale fractal features of pavement texture and proposed an interpretable machine learning model for skid resistance evaluation based on these features. The following conclusions were drawn from the experimental results:

(1) A multi-scale fractal analysis of the 3D texture of asphalt pavement was carried out. It was found that the fractal dimension of AC-13 is relatively concentrated, that of OGFC-10 is in between, and that of SMA-16 is the most dispersed. At different decomposition levels, the mean value of the fractal dimension *D* shows a similar trend: it first increases with the number of decomposition levels and then decreases to approach 2. For the three types of gradations, the multifractal parameter ∆α changes in the opposite way to the fractal dimension *D*. With an increase in the number of decomposition levels, ∆f shows a general downward trend.
(2) A skid resistance evaluation model based on CMA-ES optimization, namely CMA-ES-XGBoost, was proposed. Compared with Linear Regression, LightGBM, and XGBoost, CMA-ES-XGBoost reduced the generalization RMSE by 36.5%, 20.3%, and 9.8%, respectively; reduced the MAE by 40.1%, 16.6%, and 10.2%, respectively; and increased R^2^ by 27.1%, 9%, and 3.4%, respectively.
(3) Based on the interpretability analysis method, the impact of each feature on the model output was intuitively explained. The five features “$D_{0.4\sim 0.8\mathrm{mm}}$”, “$D_{0.2\sim 0.4\mathrm{mm}}$”, “$D_{>25.6\mathrm{mm}}$”, “$D_{3.2\sim 6.4\mathrm{mm}}$”, and “Sand” have the greatest impact on skid resistance. Among them, the feature “$D_{0.4\sim 0.8\mathrm{mm}}$”, which is the fractal dimension of the texture in the range of 0.4 to 0.8 mm, had the most significant impact on the model output.
(4) In the analysis of feature interactions, there was a certain interaction between “$D_{0.4\sim 0.8\mathrm{mm}}$” and “$D_{0.2\sim 0.4\mathrm{mm}}$”. When the value of “$D_{0.2\sim 0.4\mathrm{mm}}$” is high, an increase in “$D_{0.4\sim 0.8\mathrm{mm}}$” tends to reduce the model’s predicted value. The value of “$D_{0.4\sim 0.8\mathrm{mm}}$” changes with the values of “$D_{>25.6\mathrm{mm}}$” and “$D_{3.2\sim 6.4\mathrm{mm}}$”. Under different combinations of values for “$D_{>25.6\mathrm{mm}}$” and “$D_{3.2\sim 6.4\mathrm{mm}}$”, there is a change in the direction and strength of the impact of “$D_{0.4\sim 0.8\mathrm{mm}}$” on the model output. In addition, when the value of “Sand” is low, the positive impact of “$D_{0.4\sim 0.8\mathrm{mm}}$” is more significant.

This study demonstrated that multi-scale fractal texture features are effective indicators for characterizing the skid resistance of pavement surfaces under sand-accumulated conditions, and the proposed CMA-ES-XGBoost model performed well in robustness experiments. When comparing traditional texture features with this paper’s multi-scale fractal texture features, it is promising to apply this paper’s multi-scale fractal features and CMA-ES-XGBoost model to in-service road pavements. However, in order to further enhance the robustness of the skid resistance evaluation model, future research directions will focus on three main areas: (1) expanding the dataset by collecting data from more gradation specimens under sand-accumulated conditions to enable more effective model training; (2) considering factors such as temperature, humidity, and pavement wear; and (3) developing a skid resistance evaluation model that combines intelligent optimization algorithms and artificial intelligence.

## Figures and Tables

**Table 1:** Specimen information.

Asphalt Mixture	Gradation Type	Number	Mass Percentage (%) Passing Through the Following Sieve Holes (mm)										
			19.0	16.0	13.2	9.5	4.75	2.36	1.18	0.6	0.3	0.15	0.075
Asphalt concrete	AC-13	3	100	100	99	83	64	46	35	25	18	14	7
Open-graded friction course	OGFC-10	3	100	100	100	95	60	16	12	10	8	6	4
Stone matrix asphalt	SMA-16	3	100	91	67	47	21	16	15	13	11	10	8

**Table 2:** Physical properties of asphalt binder.

Properties	Measured Value
Softening point (°C)	72
Ductility (15 °C, 5 cm/min, cm)	38
Penetration (25 °C, 100 g, 5 s, 0.1 mm)	55
Flash point (°C)	283

**Table 3:** Physical properties of aggregates.

Properties	Measured Value
Crushed value (%)	20.9
Los Angeles abrasion loss (%)	25.1
Hygroscopy (%)	1.42
Adhesion	6
Ruggedness (%)	10.9

**Table 4:** Distribution of sand particles.

Range (mm)	>0.5	0.25~0.5	0.075~0.25	<0.075
Percentage (%)	0	1.4	96.7	1.9

**Table 5:** Multi-scale fractal features of 3D texture.

Category	Feature Name	Description
Fractal dimension	$D_{0.1\sim 0.2\mathrm{mm}},\;D_{0.2\sim 0.4\mathrm{mm}},\;D_{0.4\sim 0.8\mathrm{mm}},$ $D_{0.8\sim 1.6\mathrm{mm}},\;D_{1.6\sim 3.2\mathrm{mm}},\;D_{3.2\sim 6.4\mathrm{mm}},$ $D_{6.4\sim 12.8\mathrm{mm}},\;D_{12.8\sim 25.6\mathrm{mm}},\;D_{>25.6\mathrm{mm}}$	Fractal dimension at each scale
Multifractal dimension	$\alpha_{0.1\sim 0.2\mathrm{mm}},\;\alpha_{0.2\sim 0.4\mathrm{mm}},\;\alpha_{0.4\sim 0.8\mathrm{mm}},\;\alpha_{0.8\sim 1.6\mathrm{mm}},\;\alpha_{1.6\sim 3.2\mathrm{mm}},\;\alpha_{3.2\sim 6.4\mathrm{mm}},\;\alpha_{6.4\sim 12.8\mathrm{mm}},\;\alpha_{12.8\sim 25.6\mathrm{mm}},\;\alpha_{>25.6\mathrm{mm}}$	Difference ∆α between maximum and minimum singularity intensities at each scale
	$f_{0.1\sim 0.2\mathrm{mm}},\;f_{0.2\sim 0.4\mathrm{mm}},\;f_{0.4\sim 0.8\mathrm{mm}},\;f_{0.8\sim 1.6\mathrm{mm}},\;f_{1.6\sim 3.2\mathrm{mm}},\;f_{3.2\sim 6.4\mathrm{mm}},\;f_{6.4\sim 12.8\mathrm{mm}},\;f_{12.8\sim 25.6\mathrm{mm}},\;f_{>25.6\mathrm{mm}}$	Difference ∆f between fractal dimensions at minimum and maximum singularity intensities at each scale

**Table 6:** Traditional texture features.

Category	Feature Name	Description
Height parameters	$S_{q}\;$(mm)	Root mean square of height
	$S_{\mathrm{sk}}\;$(/)	Skewness
	$S_{\mathrm{ku}}\;$(/)	Kurtosis
	$S_{p}\;$(mm)	Maximum height of peak
	$S_{v}\;$(mm)	Maximum height of pit
	$S_{z}\;$(mm)	Maximum height
	$S_{a}\;$(mm)	Arithmetical mean of height
Function parameters	$S_{\mathrm{mr}}\;$(%)	Areal material ratio
	$S_{\mathrm{mc}}\;$(mm)	Inverse areal material ratio
	$S_{\mathrm{dc}}\;$(mm)	Material ratio difference of height
Spatial parameters	$S_{\mathrm{al}\;}$(mm)	Auto-correlation length
	$S_{\mathrm{td}}\;$(°)	Texture direction
	$S_{\mathrm{sw}\;}$(mm)	Dominant spatial wavelength

**Table 7:** Metrics of XGBoost trained with different datasets.

Dataset	RMSE	MAE	R^2^
Height parameters	5.47	3.204	0.739
Height + function parameters	4.83	2.98	0.796
Height + function + spatial parameters	4.588	3.05	0.8157
Multi-scale fractal (ours)	4.202	3.249	0.8454

**Table 8:** Optimal hyperparameter configuration.

Hyperparameter Name	Optimal Value
max_depth	12
learning_rate	0.2225
n_estimators	80
reg_alpha	0.3079
reg_lambda	0.7342
gamma	0.6779
min_child_weight	13
colsample_bytree	0.8213
subsample	0.7903

**Table 9:** Quantitative comparison of skid resistance evaluation models.

Model	RMSE	MAE	R^2^
Linear Regression	5.971	4.869	0.6878
LightGBM	4.753	3.496	0.8022
XGBoost	4.202	3.249	0.8454
CMA-ES-XGBoost	3.789	2.916	0.8743

## Data Availability

Some or all data, models, or code that supports the findings of this study is available from the corresponding author upon reasonable request.

## Abbreviations

The following abbreviations are used in this manuscript:

3D	Three-dimensional
AC-13	Asphalt concrete with a maximum nominal particle size of 13 mm
OGFC-10	Open-graded friction course with a maximum nominal particle size of 9.5 mm
SMA-16	Stone matrix asphalt with a maximum nominal particle size of 16 mm
XGBoost	eXtreme Gradient Boosting
LightGBM	Light Gradient Boosting Machine
CMA-ES	Covariance Matrix Adaptation Evolution Strategy
SHAP	SHapley Additive exPlanations
LIME	Local Interpretable Model-agnostic Explanations
GESD	Generalized Extreme Studentized Deviate
NRIA	Neighboring-region Interpolation Algorithm
TDFA	Tire–pavement Dynamic Friction Analyzer
CFME	Continuous Friction Measurement Equipment
HHT	Hilbert–Huang Transformation
DFT60	Test value of Dynamic Friction Tester at 60 km/h
MTD	Mean texture depth
MPD	Mean profile depth
BPT	British Pendulum Tester
RMSE	Root-mean-square error
MAE	Mean absolute error

## References

[1] Anupam K., Tang T., Kasbergen C., Scarpas A., Erkens S. (2021). 3-D thermomechanical tire–pavement interaction model for evaluation of pavement skid resistance. Transp. Res. Rec..

[2] Kogbara R.B., Masad E.A., Kassem E., Scarpas A., Anupam K. (2016). A state-of-the-art review of parameters influencing measurement and modeling of skid resistance of asphalt pavements. Constr. Build. Mater..

[3] Riahi E., Edjeou W., Buisson S., Gennesseaux M., Do M.T. (2022). Estimation of water depth on road surfaces using accelerometric signals. Sensors.

[4] Song L., Yun D., Ye W., Gao J. (2024). Characteristics of open-graded friction course macrotexture and macrostructure and its effect on skid resistance under rainfall. Materials.

[5] Zhu X., Yang Y., Zhao H., Jelagin D., Chen F., Gilabert F.A., Guarin A. (2021). Effects of surface texture deterioration and wet surface conditions on asphalt runway skid resistance. Tribol. Int..

[6] Chu L.J., Fwa T.F. (2018). Pavement skid resistance consideration in rain-related wet-weather speed limits determination. Road Mater. Pavement Des..

[7] Dan H.C., He L.H., Xu B. (2017). Experimental investigation on skid resistance of asphalt pavement under various slippery conditions. Int. J. Pavement Eng..

[8] Permanent International Association of Road Congresses (PIARC) Report of the Committee on Surface Characteristics. Proceedings of the XVII World Road Congress.

[9] Kienle R., Ressel W., Götz T., Weise M. (2020). The influence of road surface texture on the skid resistance under wet conditions. Proc. Inst. Mech. Eng. Part J J. Eng. Tribol..

[10] Du Y., Liu C., Song Y., Li Y., Shen Y. (2019). Rapid estimation of road friction for anti-skid autonomous driving. IEEE Trans. Intell. Transp. Syst..

[11] Xie T., Yang E., Chen Q., Rao J., Zhang H., Qiu Y. (2024). Separation of macro-and micro-texture to characterize skid resistance of asphalt pavement. Materials.

[12] Wang W., Liu B., Jin D., Yu M., Zeng J. (2024). Durability Investigation of Ultra-Thin Polyurethane Wearing Course for Asphalt Pavement. Materials.

[13] Dong Y., Wang Z., Ren W., Jiang T., Hou Y., Zhang Y. (2023). Influence of morphological characteristics of coarse aggregates on skid resistance of asphalt pavement. Materials.

[14] Yang G., Wang K.C., Li J.Q., Wang G. (2022). A novel 0.1 mm 3D laser imaging technology for pavement safety measurement. Sensors.

[15] Li Y., Qin Y., Wang H., Xu S., Li S. (2022). Study of texture indicators applied to pavement wear analysis based on 3D image technology. Sensors.

[16] Díaz-Torrealba R., Marcobal J.R., Gallego J. (2024). Modelling Asphalt Overlay As-Built Roughness Based on Profile Transformation—Case for Paver Using Automatic Levelling System. Sensors.

[17] Chu C., Wei Y., Wang H. (2023). Improved 3D pavement texture reconstruction method based on interference fringe via optimizing the post-processing method. Sensors.

[18] Yang G., Wang K.C., Li J.Q. (2021). Multiresolution analysis of three-dimensional (3D) surface texture for asphalt pavement friction estimation. Int. J. Pavement Eng..

[19] Yu W., Li J.Q., Yang G., Wang K.C., Attoh-Okine N. (2022). Hilbert-Huang transformation (HHT) based texture profile analysis for continuous friction characterisation of pavements. Int. J. Pavement Eng..

[20] Li F., Ablat G., Zhou S., Liu Y., Bi Y., Weng Z., Du Y. (2021). 2D-wavelet based micro and macro texture analysis for asphalt pavement under snow or ice condition. J. Infrastruct. Preserv. Resil..

[21] Liu C., Zhan Y., Deng Q., Qiu Y., Zhang A. (2021). An improved differential box counting method to measure fractal dimensions for pavement surface skid resistance evaluation. Measurement.

[22] Miao Y., Song P., Gong X. (2014). Fractal and multifractal characteristics of 3D asphalt pavement macrotexture. J. Mater. Civ. Eng..

[23] Ran M.P., Xiao S., Zhou X.L., Xiao W.X. (2018). Evaluation of segregation in asphalt pavement surface using concave multifractal distribution. J. Test. Eval..

[24] Ribeiro M.T., Singh S., Guestrin C. “Why should I trust you?” Explaining the predictions of any classifier. Proceedings of the 22nd ACM SIGKDD International Conference on Knowledge Discovery and Data Mining.

[25] Scott M., Su-In L. (2017). A unified approach to interpreting model predictions. Adv. Neural Inf. Process. Syst..

[26] Kováč M., Brna M. (2021). The influence of the pavement surface texture on the Pendulum Test Value. IOP Conf. Ser. Mater. Sci. Eng..

[27] (2017). Chinese Communications, Specifications for Design of Highway Asphalt Pavement.

[28] (2011). Chinese Communications, Standard Test Methods of Bitumen and Bituminous Mixture for Highway Engineering.

[29] (2019). Chinese Communications, Field Test Methods of Highway Subgrade and Pavement.

[30] Van de Wouwer G., Scheunders P., Van Dyck D. (1999). Statistical texture characterization from discrete wavelet representations. IEEE Trans. Image Process..

[31] Chen T., Guestrin C. Xgboost: A scalable tree boosting system. Proceedings of the 22nd ACM SIGKDD International Conference on Knowledge Discovery and Data Mining.

[32] Ke G., Meng Q., Finley T., Wang T., Chen W., Ma W., Ye Q., Liu T. (2017). Lightgbm: A highly efficient gradient boosting decision tree. Adv. Neural Inf. Process. Syst..

[33] Akiba T., Sano S., Yanase T., Ohta T., Koyama M. Optuna: A next-generation hyperparameter optimization framework. Proceedings of the 25th ACM SIGKDD International Conference on Knowledge Discovery & Data Mining.

